# Forensic Medicine

**Published:** 1907-05

**Authors:** E. S. McKee

**Affiliations:** Cincinnati


### PROGRESS IN MEDICAL SCIENCE.

### Forensic Medicine.

Reported by E. S. McKee, M.D., Cincinnati.

### CRIMINAL RESPONSIBILITY OF THE INSANE.

Dr. F. E. Daniel, editor of the Texas Medical Journal, discusses this subject *in extenso* in his journal. He says that there is a wide-spread and growing conviction that the reform of the criminal law is a pressing necessity. The inefficiency of our penal statutes is due to the fact that they are made by men who, for the most part, make no pretensions to scientific knowledge and are notoriously adverse to being advised. The unwise practice in some states of paying legislators day wages is, in a measure, responsible for this. Such pay is not calculated to command a high order of law making talent. That the jurisprudence of insanity is far behind the present status of medical science on this subject is very generally admitted; it belongs to a past age and is therefore not adapted to the needs of a latter-day civilisation. The crude division into idiots and lunatics of two centuries ago survives in jurisprudence today. What other course than that of medical science should our law makers follow in legislating upon a subject better understood by physicians than by any other class of investigators? There are forty-four forms of insanity known to alienists.

From the standpoint of the medical jurist the jurisprudence of insanity is defective in at least three particulars: First—the defendant in a murder trial has not the benefit of a diagnosis by the light of modern science, because recent discoveries and conclusions of medical science are not comprehended in the existing system. The laws have not been made to conform thereto, nor do the courts permit textbooks, the standard authorities, to be quoted in support of alleged insanity. Second—the law leaves to the determination of a jury, often of unenlightened men, metaphysical questions that baffle the ablest scientific minds, to wit, the existence or non-existence of insanity, the degree of impairment of free will, and the extent of responsibility of a person adjudged insane by medical experts. Third—the courts do not exercise proper discrimination in allowing medical men to pose as experts. There should be a medical court in every state, paid by the people, to whom should be left the adjudication of all points of medicine in its relation to law, just as we have courts of law to settle all legal points. Trial by jury is a relic of barbarous ages, and has degenerated in a large number of instances into a travesty of justice. If accused of a crime I had rather trust my fate to the toss up of a penny, than to stand trial by a jury to whom is given the determination of questions so far beyond their comprehension.

Our penal system is based upon the ancient law "an eye for an eye and a tooth for a tooth." Vengeance seems to be the chief end,—retaliation rather than justice. Our system of jurisprudence should not only be humane, it should be intelligent. The protection of society, the deterring of criminals, and the lessening of crime are the ostensible objects of capital punishment. It is a demonstrated failure. The ends can be secured by means less revolting. I am well aware that the hope of instituting radical changes in a system so universal and so long established is Utopian; but were everybody content with the existing condition of things there would be no progress in any department of human activity—in law, medicine, art, science, literature, finance or commerce. No errors would be corrected, no evils eradicated. Hence, when human life so often depends upon rules of the court based upon an antiquated conception of insanity, it is needful to insist that the voice of science shall be heard, and that the great truths revealed by the laborious investigation and experimentation, truths vital to the dearest interests of mankind, shall be utilised in medical and criminal jurisprudence. Our system needs to be remodeled, made more comprehensive, and adapted to the changed condition of the knowledge of insanity and to the demands of an advanced civilisation.

---

### SUCCESSFUL SOOTHING SYRUPS.

THE journal of the American Medical Association, February 9, 1907, reports the following: Death of a child of 10 months from Mrs. Winslow's soothing syrup reported by Dr. John E. Campbell, South St. Paul, Minn. The death of a child from Rex cough syrup is reported by Dr. Thos. C. Buxton, Decatur, Ill., coroner of Macon County. Dr. J. Elliott Dorn, Brooklyn, reports the death of a child from Monell's teething syrup. Dr. Jesse R. Cooper, New Castle, Pa., reports the death of the twin children of Joseph Minolich, of New Castle, Pa., from opium poisoning, the result of taking Kopp's Baby's Friend. The father was agent for the preparation.

---

### WAIVER OF PRIVILEGED COMMUNICATION BY HEIR.

IN the case of Sibley vs. Morse, Supreme Court of Michigan, a physician was called to testify as to the mental condition of testator at the time two codicils were drawn to his will. On cross-examination the physician was asked as to his having treated testator for urethritis. Excluded on objection. The contention was then raised that testator's son, as heir-at-law, might waive the statute protecting the patient from the discovery of facts learned by the physician while treating him. But whether the heir can, against the protest of legal representatives (as executors, etc.,) waive the right, the court does not decide, on the ground that it was not necessary to do so in this case. It says, however, that there is authority supporting the contention that he may do so. (Thompson vs. Ish 99 Mo. 160). The reason given why it was unnecessary to decide the question here was that in any view of the case the court thinks the testimony in question was too remote to be of any value in determining the mental condition of the testator a number of years later, and no error was committed in its exclusion which prejudiced the rights of the contestant.

---

### CONSENT TO OPERATION.

THE supreme court of Illinois has had this subject before it recently in the case of Pratt vs. Davis. The result of this trial emphasises the necessity of surgeons having a clear understanding of their legal liabilities in undertaking important operations, and the prudence of requiring explicit consent of the patient or his legal representative before beginning an operation. The decision covers three points of much interest to surgeons: First—what is sufficient consent to an operation? Second—How much is implied in consent once given? Third—What is the privilege and duty of the surgeon in emergencies arising in the course of an operation undertaken with previously obtained consent?

The case in question was as follows: patient, married, aged 40, had suffered from epileptic seizures for 15 years; came with her husband to a sanitarium for treatment. There was found laceration of the womb and other conditions which required surgical interference. She was operated on for these conditions and sent home but was not improved. She was then told to come back to the sanitarium for further operation. The surgeon then removed her womb without expressly telling her that he was going to do so or gaining her consent or that of her husband, though the latter was implied.

The patient was no better but rather grew worse and later was adjudged insane and sent to an asylum. Suit for damages was instituted for removing the uterus without consent, and a verdict for three thousand dollars given. The Supreme Court affirmed the decision of the Appellate Court. The decision of the court on the third point is of importance; for it tends to put the duty of the surgeon in the course of an operation already undertaken in a clearer light. It is the duty and legal right of the surgeon in the presence of unexpected complications, arising in the course of an operation, to use his highest skill and judgment even if the consent of the patient or his representatives can not be obtained. It is also the right and duty of the surgeon to act in accordance with the best teachings of surgery in emergencies in which consent cannot be obtained, even to the extent of performing operations.

---

### FALSE PRETENSES IN PROMISING IMPOSSIBLE CURES.

A FARMER suffering from epileptic fits was promised a sure cure by a firm treating by electricity. He was told that it was a lucky thing for him that he came when he did, that he would have been beyond the reach of relief in another month. The ordinary cost of the treatment was one hundred pounds, but as he was a poor man they would do it for forty pounds. Of this ten pounds was paid down, but no treatment was given. He afterward learned of the impossibility of a cure from his condition, idiopathic epilepsy, and sued for the return of the money. The court ruled that by guaranteeing to do something which was impossible, the firm had obtained money under false pretenses, and a verdict was given for the money with costs.—Australasian Medical Gazette.

---

### MAL-PRACTICE SUIT ENDED.

IN the case of Mrs. Hixon vs. Dr. John W. Rabe, Akron, O., the jury returned a verdict for the defendant on technical grounds without leaving the box, in accordance with the instructions of the court.

---

### TO STOP QUACKERY.

A COMMITTEE has been appointed by Dean Remington, of the Philadelphia College of Pharmacy, to frame a bill to go before the Pennsylvania legislature to secure the passage of a pure food and drug law, The provisions of the bill are to be similar to that enacted by the federal government. The movement was inaugurated by the druggists and physicians of Philadelphia.

---

### PHYSICIANS TO REPORT ACCIDENTS.

WISCONSIN has a law requiring physicians to report accidents, as the state is desirous of classifying accidents. The statistician believes that physicians are very negligent in making these reports.

### VARIOUS VALUES OF PHYSICIANS' SERVICES.

THE Supreme Court of Colorado discusses the criteria governing the value of medical services. The value of professional services may depend considerably on the character and standing of him who performs them. Diversities of gifts, period of time passed in the profession, the experience, degree of skill and faculty of using professional knowledge, should be considered. The services of some are worth more than the services of others, because they will command more. Evidence of professional standing is clearly admissible. The fact that a doctor is very busy tends to show his professional standing. If constant practice in the art of his profession renders a practitioner more capable than he otherwise would be, the extent of such practice is a matter which may be properly inquired into for the purpose of determining the value of the services.

### A RESPONSIBLE LUNATIC.

A CITIZEN of Kentucky was recently placed on trial by a jury at his own request and sent to the asylum on his own testimony. He said that he had distinct impulses to kill his wife and children and burn his house and then kill himself. He feared these impulses would be so strong that he would be unable to resist them, hence his application to the court to be sent to the asylum, which request was quickly granted. He manifested great joy that he was to be restrained in such a manner that his wife and children would be safe. Such a case as this raises the point as to accountability, particularly as to the right and wrong test. If this man had killed his family he would have been held legally accountable under certain applications of the right and wrong test, which holds that if the man knows the nature of and quality of the act and knows that it is wrong, then he is to be punished. The fact that he recognised the act as wrong showed that he was rational, hence the court committed a man to the asylum who was rational, a gross miscarriage of justice, though it possibly saved lives.

### BODY EXHUMED AS A RESULT OF UNFOUNDED ATTACK ON PHYSICIAN.

SOME eight months after the death and interment of a widow, aged 63, in London, the brother of the deceased made affidavit that the physician was in a great hurry to get the body buried. He also said that he believed that the death of his sister was due either to the administration of improper drugs or to incorrect treatment. At the inquest which was held the doctor stated that he had first treated the woman for dyspepsia in 1898. Three years later symptoms of cardiac degeneration set in. In 1904 Dr. Rose Bradford saw the patient with him and concurred entirely with the treatment. As to the funeral, he had nothing to do with it. Evidence was given by Dr. Wilcox of the home office analysis, and Dr. F. J. Smith, professor of Forensic Medicine at the London Hospital, that the body showed no evidence at all of poisoning. The jury rendered a verdict that the certificate given by the doctor was correct and that there were no grounds for the imputations made.

---

### THE PATRICK CASE.

THE Patrick case in New York (*Wisconsin Medical Recorder*) is still attracting considerable attention. Mr. Wm. M. Rice, aged 84, died from what his attending physician ascribed as natural causes. Within two hours after his death the body was embalmed with "Falcon embalming fluid, which contains formaldehyde gas in solution. The autopsy forty-one hours later and following was found left lung congested and edematous; the right lung the same, and a small area of consolidated lung tissue about the size of a twenty-five cent piece in lower lobe." The prosecution claimed that this congestion of lungs was due to chloroform administered by Patrick. Expert testimony was arrayed on both sides. The Medico-Legal Society of New York became so interested in the subject that they appointed a committee to report on the case. One of the points that they were to report on was: "Could the injection of this embalming fluid two hours after death, before rigor mortis set in, cause this congestion of the lungs?" They answered that it could not, but it could cause a condition that could not be differentiated from congestion with the naked eye. The medical testimony cannot be reviewed in the Supreme Court but it is within the province of the governor to appoint a commission to report on the case.

